# Electrostatic Aggregation of Charged, Polarizable Particles in Extreme Atmospheric Environments

**DOI:** 10.1021/acs.jpca.5c02515

**Published:** 2025-08-05

**Authors:** Cameron P. Reeve, Connor Williamson, Evan Shelton, Anthony J. Stace, Elena Besley

**Affiliations:** School of Chemistry, 6123 University of Nottingham, Nottingham NG7 2RD, U.K.

## Abstract

Extreme atmospheric environments are often characterized by scorching temperatures and high densities of charged, polarizable particles. This study investigates two distinctly different extreme environments, volcanic ash plumes and the Venusian atmosphere, where the influence of particle polarization plays a pivotal role in driving electrostatic aggregation, particularly through like-charge attraction at small separations, which is often neglected by conventional models. In these conditions, accounting for complex polarization effects increases the estimation of collision efficiency and collision cross section by up to 25% as well as reduces the value of the velocity critical for aggregation by up to 30%, as compared to predictions by Coulomb’s Law and the hard-sphere limit. These insights have wide-ranging implications for modeling charged particle dynamics in broader industrial, atmospheric, and astrophysical contexts.

## Graphical abstract

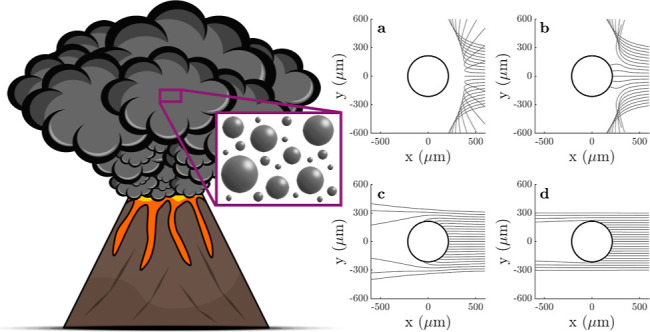

## Introduction

As extreme climate events become an increasingly regular occurrence, it becomes difficult to define what is meant by “extreme” in atmospheric science, and this term is used flexibly in the field.^1–4^ While extreme conditions affect all physical factors (pressure, pH, radiation, etc.), high-temperature atmospheric environments receive notable interest due to their link to a wide range of industrial processes.^5–8^ High-temperature research also has extensive astronomical applications from the design of thermal protection systems for spacecrafts to the studies of hot exoplanets.^9–13^

The blistering Venusian atmosphere is an example of a high-temperature extreme environment, which is thick with dense clouds of charged particles.^14–16^ While the similarity in size, mass, and composition of Venus and Earth bears promise for lifeforms, the surface of Venus appears inhospitable due to crushing pressures and scorching temperatures.^17^ As the sole material consistent with spectroscopic data, sulfuric acid, photochemically produced from sulfur dioxide and water, is widely considered to be the primary constituent of Venusian clouds (∼80% H_2_SO_4_).^18,19^ Since NASA’s fruitful Pioneer Venus missions, there has been substantial research conducted into the microphysics and morphology of Venusian clouds.^14,20,21^ More recent studies indicate that Venus’ atmosphere could potentially act as a depot for desiccated microbial life.^22^

Akin to Venusian clouds, volcanic ash plumes on Earth have garnered considerable interest^23–26^ due to their destructive history and the continuous threat they pose. The high particle density and consequent opaqueness of volcanic ash can cause major disruptions to the climate, international travel, and human health.^26–28^ Volcanic lightning, frequently observed in ash plumes during eruptions, is caused by the electrification of ash particles.^29,30^ Although the exact composition is specific to the particular volcano or even eruption, volcanic ash clouds are composed primarily of silicate minerals (50–80 wt % SiO_2_).^31^ These partially crystalline silicate particles (30–40% crystalline) readily undergo tribocharging, aggregation, and fission, generating a variety of particle sizes, charges, and surface charge densities.^32–34^

A comparison of the fundamental properties of these two extreme atmospheric environments is displayed in Table 1 using data sourced from literature.^14,18,31,32,35–38^ Both cloud systems contain particles with a radius on the micrometer scale and cannot be modeled as point charges; instead, the particles must be treated as dielectric spheres, where a dielectric material is defined as an insulator polarizable by an external electric field. The behavior of charged polarizable particles is an important consideration for several noteworthy chemical and physical phenomena. The electrostatic interactions that govern the dynamics of charged particles are fundamental in biological systems^39,40^ as well as in numerous industrial processes such as laser printing,^41^ powder coating,^42,43^ nanodiamond self-assembly,^44^ and charge scavenging.^45^ Developing a deeper understanding and advancing the methods used to describe and quantify the interactions underpinning such applications remains the subject of considerable research.^46–48^ Electrostatic frameworks taking into account particle polarization have recently been applied to study aggregation mechanisms in the atmospheres of Earth and Titan,^49,50^ but there are limited capabilities of modeling dynamics in these atmospheric environments due to the complexity of interparticle interactions.

**Table 1:** Fundamental Properties Defining the Atmospheric Environments of Volcanic Ash and Venusian Clouds^14,18,31,32,35–38^

fundamental property	volcanic ash	Venusian clouds
temperature (K)	300–1300	200–400
particle radius (μm)	20–200	0.15–4
primary constituent	SiO_2_ (50–80 wt %)	H_2_SO_4_ (∼80%)
density (g/cm^3^)	2.44–2.57	∼1.84
coefficient of restitution	0.69	0.78
dielectric constant^a^	4–12	100
phase	solid	droplet^b^

a The values of the dielectric constant depend on the specific temperature and particle composition.

b The exact phase of Venusian cloud particles remains inconclusive, particularly in the upper cloud layer.

A particularly intriguing result of incorporating polarization effects is the counterintuitive phenomenon of like-charge attraction at small separation distances.^51^ Particles with high dielectric constants (*k* ≥ 20), such as liquid droplets of water, ammonia, or methanol, are significantly more likely to coalesce than weakly polarizable particles (*k* ≈ 2), such as oils or plastics. Several other factors affect coalescence during the collision of like-charged, polarizable particles, including the charges involved, particle size, initial relative velocity, and coefficient of restitution, which reflect the elasticity of a collision. Indeed, polarization effects are much more significant when large disparities in charge or size exist between particles, with like-charge attraction much more likely to occur in these cases.

## Cross Sections for Collisions of Like-Charged, Polarizable Particles

When long-range interactions in particle dynamics are taken into account, the relative velocity of the colliding particles becomes a pivotal consideration. The critical velocities that determine coalescence can be obtained from the energy associated with a collision. The minimum initial relative velocity, *v*_rel_^min^, that two colliding like-charged particles require to overcome the repulsive Coulomb energy barrier, *E*_Coul_, is defined as^49^

1 $$v_{rel}^{\min}=\sqrt{\frac{2E_{Coul}}{\mu }}$$

where μ is the reduced mass of the two particles. The maximum initial relative velocity, *v*_rel_^max^, leading to coalescence, taking into account the kinetic energy lost in collision, as defined by the coefficient of restitution, is given by

2 $$v_{rel}^{\max}=\sqrt{\frac{2\left[\left(E_{Coul}-E_{0}\right)/{C_{r}}^{2}+E_{0}\right]}{\mu }}$$

where *E*_0_ is the electrostatic interaction energy at touching point, and *C*_r_ is the coefficient of restitution. The value of *E*_0_ can be either negative, leading to the formation of stable aggregates, or positive, leading to either metastable or unstable aggregate formation. In the case of two like-charged volcanic ash particles, a stable aggregate can be formed, and the possible outcomes of a collision are illustrated in Figure 1.

**Figure 1:**
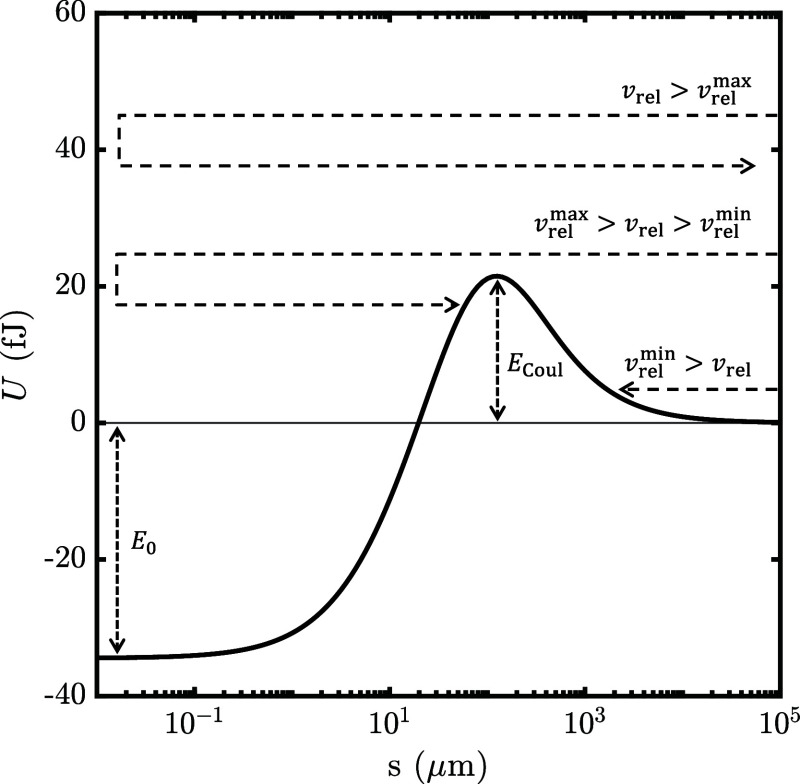
Outcomes of a collision between two like-charged volcanic ash particles (*r*_1_ = 156.7 μm, σ_1_ = 0.0473 μC/m^2^; *r*_2_ = 57.05 μm, σ_2_ = 1.74 μC/m^2^; *k* = 8). The profile (solid line) of the electrostatic interaction energy, *U*, is shown as a function of the surface-to-surface separation, *s*. Particle aggregation depends on the initial relative velocity, *v*_rel_, the energy barrier, *E*_Coul_, and the energy at contact point, *E*_0_. The latter two parameters can be used to determine the minimum (*v*_rel_^min^) and maximum (*v*_rel_^max^) initial relative velocities that would result in aggregation, following eqs 1 and 2.

Particles are initially separated to a distance where the electrostatic interaction is negligible (≤1% of *E*_Coul_), allowing for a statistical comparison to the Maxwell–Boltzmann distributions of gaseous systems at equilibrium.^52^ While this setup gives insight into the aggregation likelihood during collisions between like-charged particles, further parameters are needed to evaluate and quantify the significance of long-range interactions in dynamic simulations.

The collision cross section, σ, is a measure of the effect of long-range interactions during a collision with applications in both molecular and particulate systems.^53,54^ It is defined as

3 $$\sigma =\pi b_{\max}^{2}$$

where *b*_max_ is the maximum value of the impact parameter resulting in a collision (i.e., surfaces touching). Traditionally, cross-section calculation techniques employ the hard-sphere approximation, where long-range interactions are neglected in favor of computational cost with *b*_max_ given by the sum of the particles’ radii. This is particularly common in ion mobility spectrometry research, where molecular cross sections act as a tool for substance identification via ion mobility measurements.^55,56^ However, long-range interactions have previously been shown to have significant influence,^54,57–59^ thus underlining the necessity to consider charge and polarization effects in cross-section calculations.

Collision efficiency, CE, is a dimensionless parameter, which highlights the importance of including long-range interactions by directly and quantitatively comparing the cross section to the hard-sphere limit,^53^ and is defined by

4 $$CE=\frac{\sigma }{\pi {\left(r_{1}+r_{2}\right)}^{2}}=\frac{b_{\max}^{2}}{{\left(r_{1}+r_{2}\right)}^{2}}$$

where *r*_1_ and *r*_2_ are the particles’ radii.

In this work, collision cross sections are obtained using dynamic simulations of interacting charged particles in two extreme atmospheric environments. The induced polarization surface charge distribution on each particle has been obtained using a classical electrostatic formalism^60^ that solves for an arbitrary number of particles with defined size and dielectric constant, embedded in a homogeneous medium. Any free charge present on a particle was assumed to be uniformly distributed on its surface. Combining the multipolar expansion approach^60^ with classical particle dynamics allows for efficient study of electrostatic assembly and aggregation processes. Dynamic simulations built upon the many-body electrostatic formalism^47^ have been shown to successfully reproduce experimental results for electrostatic self-assembly of crystals^61–63^ and clustering processes in charged granular streams.^64^

In the simulations of a collision between two like-charged, polarizable particles, the larger particle is initially considered to have average kinetic energy, $\frac{1}{2}k_{B}T$ , while the initial velocity of the smaller particle is varied. Given that, at thermal equilibrium, larger particles travel significantly slower than smaller ones, as outlined in the Maxwell–Boltzmann formulation,^52^ this assumption seems reasonable. Crucially, this allows the initial relative velocity to be separated into individual particle velocities in the laboratory reference frame, so that simulations can be conducted for a range of initial relative velocities. By varying the impact parameter and observing which cases lead to collisions, *b*_max_ can be determined and the relationship between the initial relative velocity and collision cross section can be understood given eq 3. Naturally, this method requires effective sampling techniques for both the impact parameter and the initial relative velocity in order to increase the computational efficiency.

### Atmospheric Conditions

As this study is focused on quantifying the effect of long-range electrostatic interactions in particle dynamics and coalescence, other perturbations that could drive particles close to each other, such as van der Waals forces, turbulent flow, falling under gravity, and weather effects, are neglected. This also includes the initial ejection experienced by volcanic ash particles and the intense wind speeds observed in the super-rotation on Venus.^65^ The specific conditions as well as the particle properties such as composition, charge, and size of the aforementioned extreme atmospheric environments are now described in detail.

### Volcanic Ash Clouds

After volcanic ash has dispersed and settled upon the Earth’s surface, the particulates can be collected and examined, leading to insights in the variation of size and composition.^26,31,32^ By recreating the ejection of volcanic ash under laboratory conditions, Méndez Harper and Dufek^32^ observed that volcanic ash particles can vary widely in surface charge density and radius. In their study, ash samples from three different volcanic eruptions (Lawetlat’la, Tonaltepetl, and Tungurahua) were analyzed after 10 min of charging, at which point a steady state of charge can be assumed, providing insight into the tribocharging mechanisms of ash clouds. A small asymmetry in the sign of charge was observed, with 1–4% more negative charges reported than positive charges. The probability distributions of radius and surface charge density can be seen in Figure 2. Using these distributions, the necessary input parameters can be selected to investigate the nucleation of ash particles statistically and dynamically. Given the almost equal split of positive and negative particles, both like- and opposite-charged interactions were considered for this atmospheric environment. For the properties of volcanic ash (charge, surface charge density and size) analyzed by Méndez Harper and Dufek,^32^ an average distribution for each parameter was determined from the three samples.

**Figure 2:**
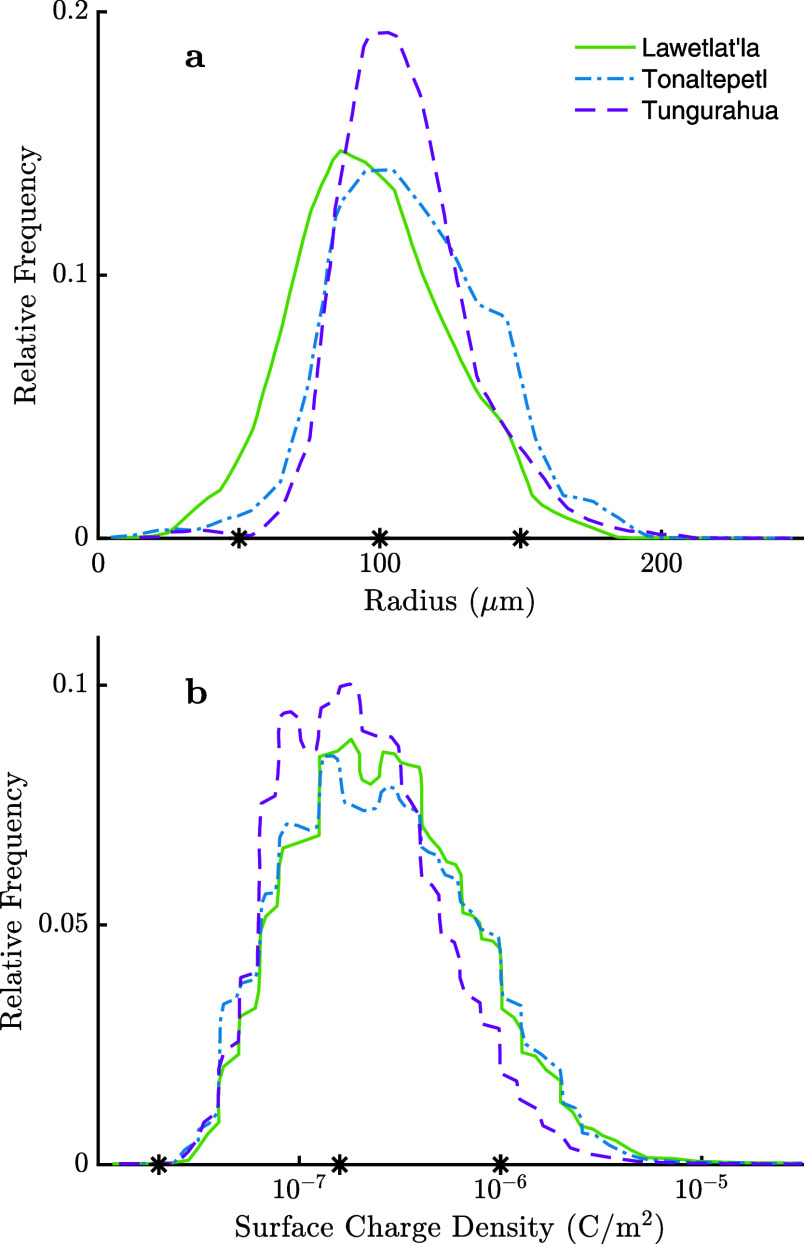
Distributions of particle size (a) and surface charge density (b) measured from three different volcanic ash samples. The values used in this study as representative of the distributions are indicated by the star marks. Reproduced from ref 32 available under a CC-BY license. Copyright 2016 Méndez Harper and Dufek.

A wide variety of trace elements can be present in volcanic ash particulates, but the exact composition is unique to each volcano and possibly even for the individual eruption. Given the exact compositions of the samples studied by Méndez Harper and Dufek,^32^ the ash particles were assigned with a corresponding dielectric constant of *k* = 8.^36,37^ Furthermore, the ash particles were modeled as malleable solid spheres, given their low coefficient of restitution (*C*_r_ = 0.69), micrometer-scale size, and high silicate composition.

### Venusian Clouds

The clouds that perpetually shroud Venus constitute the largest aerosol system among the terrestrial planets.^16^ Observations from the Pioneer Venus missions^14^ indicate the main cloud deck, which extends between 47.5 km and 70 km, can be subdivided into three distinct layers, with the variation of particle size and number density summarized in Table 2. The size distributions of particles are multimodal across all cloud regions, with two size modes extending across the entire cloud deck and the third mode only present in the middle and lower cloud layers. In the upper cloud region, bimodal particle distributions of 0.2 and 1.0 μm radius were identified, whereas the middle and lower cloud regions were reported to be populated by trimodal particle distributions with radii in the range of 0.15–4.0 μm.

**Table 2:** A Summary of Venusian Cloud Properties^a^

cloud layer	altitude (km)	temperature (K)	mean radius (μm)	average number density (cm^–3^)
upper	56.5–70.0	286–225	0.2, 1.0 (bimodal)	1500, 50
middle	50.5–56.5	345–286	0.15, 1.25, 3.5 (trimodal)	300, 50, 10
lower	47.5–50.5	367–345	0.2, 1.0, 4.0 (trimodal)	1200, 50, 50

a The mean radius is calculated as the average particle size across the altitude range for each cloud layer. Reproduced from ref 14 available under a CC-BY license. Copyright 1980 Knollenberg and Hunten.

In this article, it is assumed that the composition of the droplets is purely H_2_SO_4_ since trace contaminants are yet to be completely identified. While the exact phase of the Venusian cloud particles remains unknown, particularly at higher altitudes, the aerosol is commonly referred to as a “droplet”,^38^ and as such, the particles are modeled as liquid spheres with a dielectric constant of *k* = 100, which is characteristic of pure sulfuric acid at room temperature. As the atmosphere of Venus is predominantly composed of CO_2_,^66^ the dielectric constant of the medium is taken to have a value of *k*_m_ = 1.26. The coefficient of restitution is taken to have the value of *C*_r_ = 0.78, based on simple calculations of the dynamics of bubbles repulsion.^67^

Galactic cosmic rays ionize neutral molecules in the atmosphere, generating high concentrations of ions and mobile electrons, which readily attach to cloud particles.^15^ While the values for exact charge and surface charge density distributions do not exist for Venusian clouds as they did for volcanic ash plumes, the variation of mean charge per particle with altitude has been calculated by Michael et al.^15^ This is shown alongside the variation of temperature with the altitude in Figure 3.

**Figure 3:**
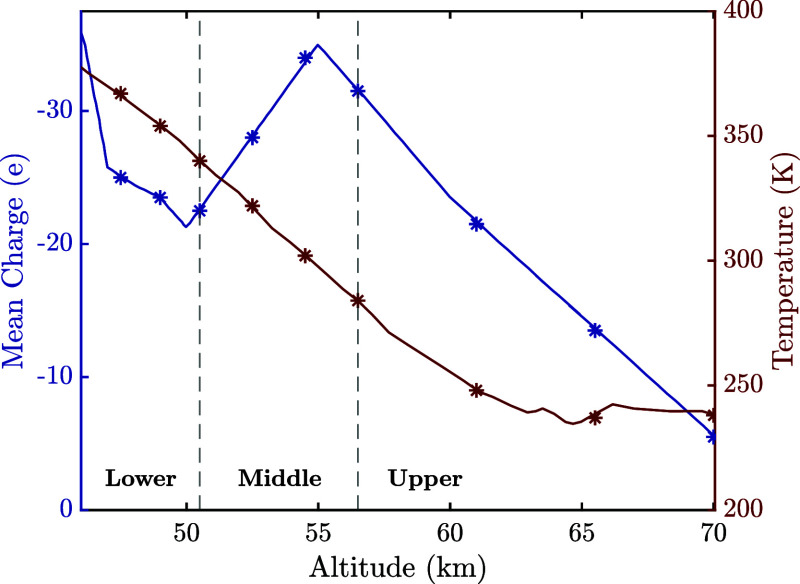
Mean charge per particle and temperature for varying altitudes across the main cloud deck of the Venusian atmosphere. The values for the altitude, mean charge, and temperature used in this study are indicated by the star marks. Reproduced from ref 14 available under a CC-BY license. Copyright 1980 Knollenberg and Hunten and from ref 15 available under a CC-BY license. Copyright 2009 Michael et al.

## Methodology

In this work, a numerical method^48,60^ was employed to compute the electrostatic interaction energy and forces between multiple charged polarizable spheres embedded within a homogeneous medium. The underlying theory is based on a boundary integral equation formulation, which uses a Galerkin approximation to attain the solution. Combining this framework with a method for solving the classical equations of motion allows for the time evolution of the system to be analyzed.^47^ In this way, the trajectories of charged, polarizable particles could be determined by using Verlet integration^68^ in conjunction with the aforementioned electrostatic framework.^48,60^ At the start of each simulation, particles were assigned an initial position and velocity, and by variation of these for each simulation, the effect of the initial relative velocity on the collision cross section could be evaluated. The cross-sectional calculations require parameters for only two particles. As Figures 2 and 3 and Table 2 highlight the vast range of possible particle pair combinations, the appropriate selection of parameters becomes important.

## Results and Discussion

The approach to examining the aggregation of charged, polarizable particles defined by Baptiste et al.^49^ was applied to two extreme atmospheric environments with an initial focus on the coalescence of like-charge particles. As the mean free path of a larger particle is much shorter than that of a smaller particle, tribocharging of larger particles is often assumed to occur to a greater extent, leading to larger particles possessing a higher average surface charge density.^69,70^ To test this assumption, which is representative of a typical system, larger particles were first assigned with larger values of the surface charge density. In the case of volcanic ash plumes, the values initially selected to be representative of the distributions are shown in Figure 2, with the surface charge density used to scale for the overall charge on a particle (*r*_1_ = 50 μm, σ_1_ = 0.02 μC/m^2^; *r*_2_ = 100 μm, σ_2_ = 0.1585 μC/m^2^; *r*_3_ = 150 μm, σ_3_ = 1 μC/m^2^). The electrostatic interaction energy was then calculated as a function of surface-to-surface separation for each pair of particles, dissimilar in size, to produce the interaction energy profiles (Figure S1 of the Supporting Information). All profiles had a local minimum at close separation distance, indicating that these particles could, in principle, experience like-charge attraction, aggregate, and form either stable or metastable state. Volcanic ash particles are subject to a range of temperatures from the moment of eruption (estimated to be ∼1300 K) to the temperature of the ground upon deposition (∼300 K). By considering the Maxwell–Boltzmann distribution of the relative velocity at this range of temperatures, the percentage of particles that could overcome the Coulomb barrier and aggregate was found to be close to zero, even for the highest temperature. Note that other perturbations that could drive these particles closer to the region of like-charge attraction (initial ejection, turbulent flow, falling under gravity, etc.) were not considered here.

Electrostatic aggregation between like charged particles in volcanic ash clouds was only predicted when the values of the radius and surface charge density were selected from the distributions in Figure 2 such that the smaller particle was assigned the higher surface charge density (*r*_1_ = 156.7 μm, σ_1_ = 0.0473 μC/m^2^; *r*_2_ = 57.05 μm, σ_2_ = 1.74 μC/m^2^). These particles form a more stable agglomerate (a deeper minimum at the contact point as shown in Figure 1) due to the highly charged small particle intensely polarizing the larger particle.

Similarly, highly charged collisions were considered for Venusian clouds, where every particle was assigned with the mean negative charge measured at each altitude, as shown in Figure 3. The corresponding electrostatic interaction energy profiles (Figure S2 of the Supporting Information) also indicate the formation of stable and metastable agglomerates, thus allowing for a statistical comparison between the critical initial relative velocities, obtained using eqs 1 and 2, and the Maxwell–Boltzmann relative velocity distribution. Our theoretical estimates of the Coulomb barrier preventing coalescence and the aggregation percentage for like-charged particles at different altitudes are shown in Table S1 of the Supporting Information. For both volcanic ash and Venusian clouds, the particle pairs with the highest minimum aggregation percentage (i.e., the percentage of particles with sufficient kinetic energy to not only collide but also coalesce through losing some of the kinetic energy upon impact) were selected to be studied dynamically.

### Dynamic Investigation

To further investigate the significance of polarization effects in electrostatic aggregation processes, the impact parameter was determined with a particle dynamics implementation of the mathematical framework derived by Hassan et al.^48^ The maximum value of the impact parameter, *b*_max_, for two like-charged, polarizable particles of volcanic ash clouds was obtained for a range of the initial relative velocities by calculating multiple trajectories of collisions. Figure 4 shows four distinct collision scenarios. First, as predicted by Coulomb’s law, if the initial relative velocity is less than the critical value (*v*_rel_ < *v*_rel_^min^, as defined by eq 1), then no collision can occur and particles strongly repel (Figure 4a). Although the particles do not collide directly (no surfaces touching), the scattering angle appears large, even for small values of the impact parameter. This signifies that the particles in volcanic ash clouds undergo various accelerations due to pure electrostatic interactions.

**Figure 4:**
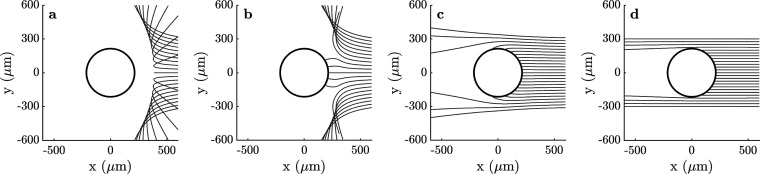
Trajectories of a small charged volcanic ash particle (*r*_2_ = 57.05 μm, *q*_2_ = 70.9 fC) approaching the larger particle (*r*_1_ = 156.7 μm, *q*_1_ = 14.6 fC) at the initial relative velocity of (a) 5 mm/s, (b) 5.2 mm/s, (c) 11 mm/s, and (d) 65 mm/s, for which *v*_rel_^min^ = 5.1 mm/s. The circle represents the combined radius of the two particles.

As the initial relative velocity is increased (*v*_rel_ ≥ *v*_rel_^min^), particles begin to collide due to polarization, as seen in Figure 4b. At first, this occurs for small values of the impact parameter, when only the central, near head-on trajectories overcome the Coulomb barrier, while the larger impact parameters lead to repulsive interactions. In this regime, as the initial relative velocity increases, so does the *b*_max_ value of the impact parameter. Even for like-charged particles, *b*_max_ can be greater than the sum of the radii of the two particles (i.e., the hard-sphere model), as depicted in Figure 4c. Since Figure 4c illustrates the largest impact parameter found for this particle pair, the initial relative velocity *v*_rel_ = 11 mm/s corresponds to the velocity at which particles are most likely to collide.

At very high relative velocities, *b*_max_ reduces to the hard-sphere approximation (CE ≈ 1 from eq 4), as demonstrated in Figure 4d. In this case, the particles behave almost independently of their charge, with only a small amount of scattering observed for particles passing close by each other. However, this scattering does highlight the strength of attractive like-charge interactions at small separations.

Given the strong effect of the initial relative velocity on *b*_max_ and the quadratic dependence of collision cross section on impact parameter (eq 3), polarization is crucial in the behavior of fast-moving particles, including their aggregation. The variation of the maximum impact parameter, collision cross section, and collision efficiency with the initial relative velocity has been investigated further for volcanic ash particles (Figure 5a–c). As seen in Figure 5, polarization effects are significant in comparison to those in a purely Coulombic model. When accounting for polarization, not only is the minimum initial velocity leading to a collision greatly reduced, by about 30% (the inset of Figure 5b), but also the maximum collision cross section is dramatically increased, by almost 20%. Notably, both the polarization and simple Coulombic models significantly deviate from the neutral hard-sphere model at low relative initial velocities, with the collision cross section for like-charged, polarizable particles being significantly greater than in nonpolarizable cases.

**Figure 5:**
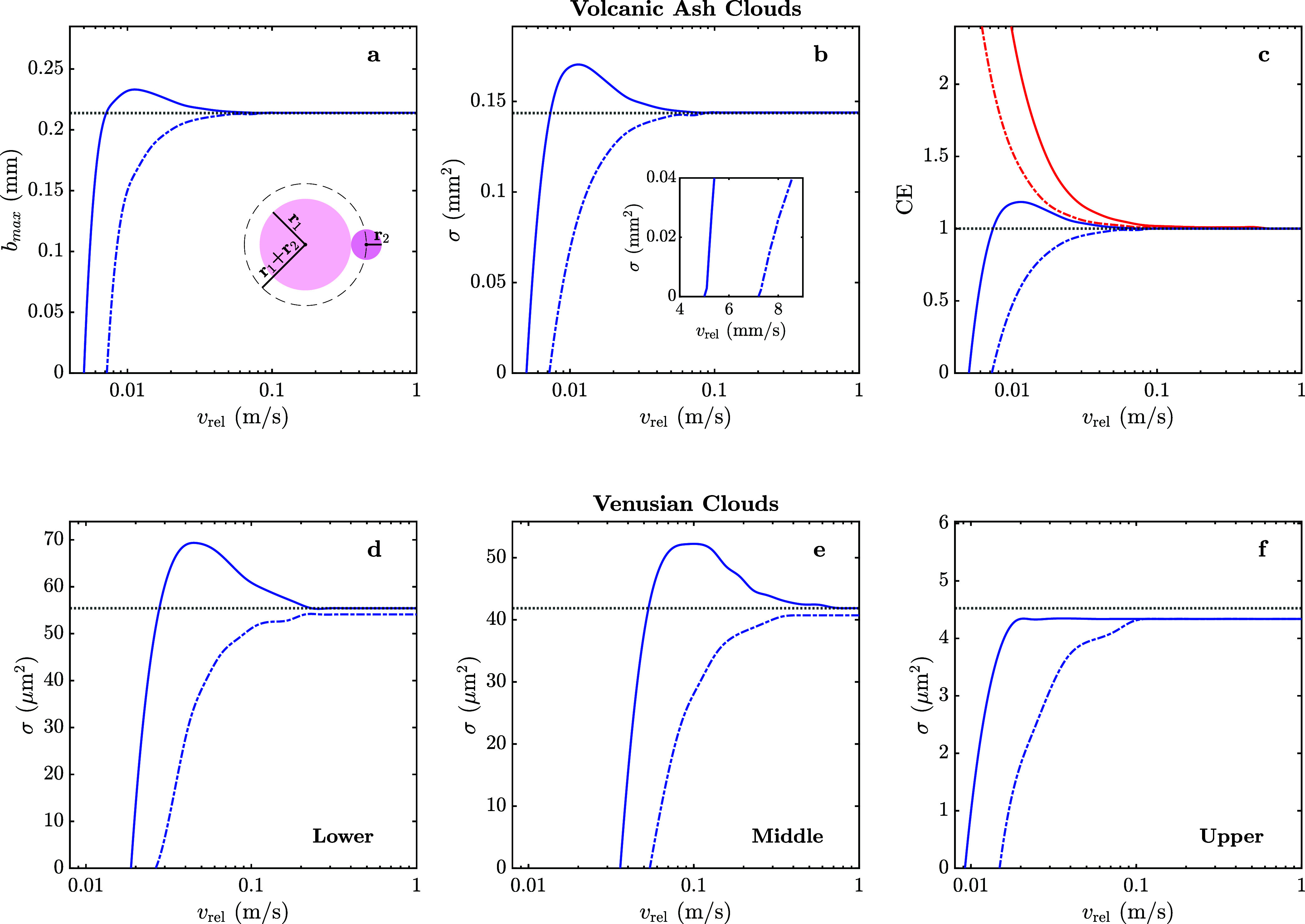
Key parameters describing a collision (i.e., surfaces touching) between charged particles in volcanic ash (top panel) and Venusian (bottom panel) clouds. For volcanic ash particles (*r*_1_ = 156.7 μm, *q*_1_ = 14.6 fC; *r*_2_ = 57.05 μm, *q*_2_ = 70.9 fC; *k* = 8), the maximum value of the impact parameter, *b*_max_, leading to a collision (a), collision cross section, σ (b), and collision efficiency, CE (c), are shown as functions of the initial relative velocity, *v*_rel_. The critical effect of particle polarization on the estimation of the collision cross section at low initial relative velocities is highlighted in the inset. For Venusian cloud particles (*k* = 100), the collision cross section in the lower (*r*_1_ = 0.2 μm, *q*_1_ = −22.5*e*; *r*_2_ = 4.0 μm, *q*_2_ = −22.5*e*) (d), middle (*r*_1_ = 0.15 μm, *q*_1_ = −28*e*; *r*_2_ = 3.5 μm, *q*_2_ = −28*e*) (e), and upper (*r*_1_ = 0.2 μm, *q*_1_ = −5.5*e*; *r*_2_ = 1.0 μm, *q*_2_ = −5.5*e*) (f) cloud layers is shown as a function of the initial relative velocity. Blue: like-charged particles; red: oppositely charged particles; solid line: polarizable particles; dashed line: nonpolarizable particles (pure Coulomb case); black dotted line: neutral hard-sphere limit.

As volcanic ash clouds contain high concentrations of both positively and negatively charged particles, the effect of polarization on two oppositely charged interacting particles was also considered, as shown in Figure 5c, where a change in sign was applied to the smaller particle. Accounting for polarization in this case led to a very significant increase in the collision cross section and thus collision efficiency by up to 70%. As expected, the maximum collision efficiency for a pair of oppositely charged particles occurs at zero initial relative velocity since there is no Coulomb energy barrier to overcome in this case. Figure 5c also distinctly illustrates that at high initial relative velocities, the collision efficiency decays to CE = 1, in line with the hard-sphere model predictions, even for oppositely charged, polarizable particles. By considering the role of polarization in volcanic ash clouds, it can be seen that nucleation could readily occur for both like-charged and oppositely charged particles at a far greater rate than the Coulombic or hard-sphere models would suggest.

Another studied atmospheric environment, the main cloud deck of Venus, is characterized by high concentrations of charged sulfuric acid particles and the high temperatures observed in its lower layer (Figure 3). The relationship between collision cross section and the initial relative velocity is displayed in Figure 5d–f for a particle pair from each of the cloud layers. While particle polarization leads to an increased collision cross section compared to simple Coulombic behavior for each layer, there is a marked difference between cloud layers. In the upper cloud region, the collision cross section reaches its maximum just below the neutral hard-sphere limit, whereas the middle and lower cloud regions both have collision cross sections in excess of the hard-sphere limit and by up to 25% greater for a range of initial relative velocities. This indicates that particles with larger charges can achieve a higher maximum collision cross section even for like-charged particles, which further emphasizes the importance of considering polarization effects for highly charged systems. It should be noted that the smoothness of the curves in Figure 5d–f is affected by the discrete sampling of the impact parameter, which also results, in some instances, in the values of the cross section not reaching the hard-sphere limit. This becomes more pronounced in the case of the Venusian atmosphere, where particles with the higher values of the dielectric constant require stricter convergence criteria,^51^ and computations are significantly more expensive.

From the minimum and maximum initial relative velocities displayed in Table 3, the approximate range of relative velocities for which particles will collide and coalesce can be deduced. The range of velocities is the widest for particle pairs in the lower and middle atmospheres of Venus, both of which had collision cross sections larger than those predicted by the neutral hard-sphere model. In the uppermost layer of Venus’ atmosphere, most energetically favorable collisions appear to be head-on.

**Table 3:** Critical Values of the Initial Relative Velocity, *v*_rel_, Obtained from the Particle Dynamics Simulations, *v*_dyn_^min^, Compared with the Values Extracted Directly from the Energy Profiles, *v*_elec_^min^ and *v*_elec_^max^

atmospheric environment	charge (*e*)		radius (μm)		relative velocity *v*_rel_ (mm/s)		
	*q*_1_	*q*_2_	*r*_1_	*r*_2_	*v*_elec_^min^	*v*_dyn_^min^	*v*_elec_^max^
volcanic ash cloud	9.1 × 10^4^	4.4 × 10^5^	156.7	57.05	5.0	5.1	10
Venusian upper cloud	–5.5	–5.5	0.20	1.0	9.2	9.6	14
Venusian middle cloud	–28	–28	0.15	3.5	38	40	120
Venusian lower cloud	–22.5	–22.5	0.20	4.0	19	19	54

The consistency of the presented results was analyzed by comparing the critical values of the initial relative velocity *v*_rel_ predicted directly from the electrostatic interaction energy profiles with the values obtained via dynamic simulations, as shown in Table 3. Immediately, it is clear that there is strong alignment between the electrostatic and dynamic picture in terms of the minimum initial relative velocity. The dynamically obtained values (*v*_dyn_^min^) fall within 5% of the corresponding electrostatic values (*v*_elec_^min^), with uncertainty arising from the discrete nature of the discontinuous sampling for the initial relative velocity and impact parameter. If the sampling intervals of relative velocity were reduced to an infinitesimally small value, the predicted value of *v*_dyn_^min^ should converge to the *v*_elec_^min^ value. Refined sampling methods and computational efficiency would allow for reduced sampling intervals and time-steps to be used in dynamic simulations. Furthermore, both approaches are limited by the termination of the multipolar expansion, as seen in Figure S3 of the Supporting Information.

## Conclusions

A general method for calculating the collision cross section of two charged dielectric particles via dynamic simulations has been presented. It has been shown that the selection of the appropriate electrostatic model is directly linked to the dynamics of the particle pair. For collisions at high initial relative velocities, the hard-sphere approximation is sufficient, but at lower initial relative velocities, a more rigorous treatment is necessary to account for the long-range particle interactions. When considering a like-charged pair, Coulomb repulsion is sufficient to describe the interaction of two particles with initial relative velocity below the critical value of *v*_rel_^min^.

In summary, considering particle dynamics in conjunction with accurate calculations of the electrostatic interaction energy and the polarization of surface charge leads to a much improved estimation of the collision cross section, which is a crucial parameter when studying the aggregation of charged particles in extreme environments.

## Supplementary Material



## Notes

Electrostatic aggregation of charged, polarizable particles in extreme atmospheric environments, which includes electrostatic interaction energy profiles, additional statistical results for the Venusian cloud system, and the graphical convergence of the multipolar expansion method (PDF)
