# Supplementary material for: Electrostatic Aggregation of Charged, Polarizable Particles in Extreme Atmospheric Environments
Source: J Phys Chem A. 2025 Aug 5;129(32):7461–9. doi: 10.1021/acs.jpca.5c02515 (PMC12359109; doi:10.1021/acs.jpca.5c02515)
Supplement: Supplementary file 1 [file jp5c02515_si_001.pdf]

Supporting Information: Electrostatic Aggregation of Charged, Polarizable Particles in Extreme Atmospheric Environments

Cameron P. Reeve,[†,‡] Connor Williamson,[†,‡] Evan Shelton,[†] Anthony J. Stace,[†] and Elena Besley*[,†]

† *School of Chemistry, University of Nottingham, Nottingham, United Kingdom, NG7 2RD, UK*

‡ *These authors contributed equally to this work.*

E-mail: elena.besley@nottingham.ac.uk

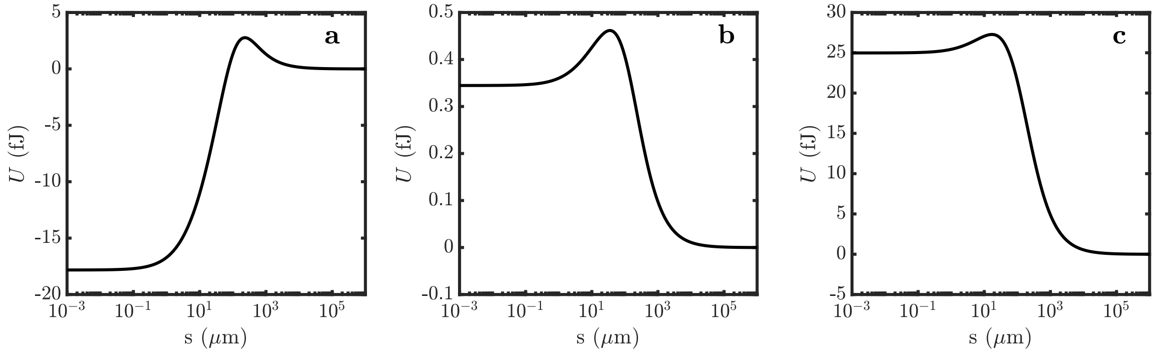

Figure S1: Profiles of the electrostatic interaction energy, U, against the surface-to-surface separation, s, for the (a) smallest-largest, (b) smallest-median, and (c) median-largest volcanic ash particle pairs ($k = 8$). The particles had the following properties: (smallest) $r_1 = 50$ µm and $\sigma_1 = 0.02$ µC/m^2, (median) $r_2 = 100$ µm and $\sigma_2 = 0.1585$ µC/m^2, and (largest) $r_3 = 150$ µm and $\sigma_3 = 1$ µC/m^2.

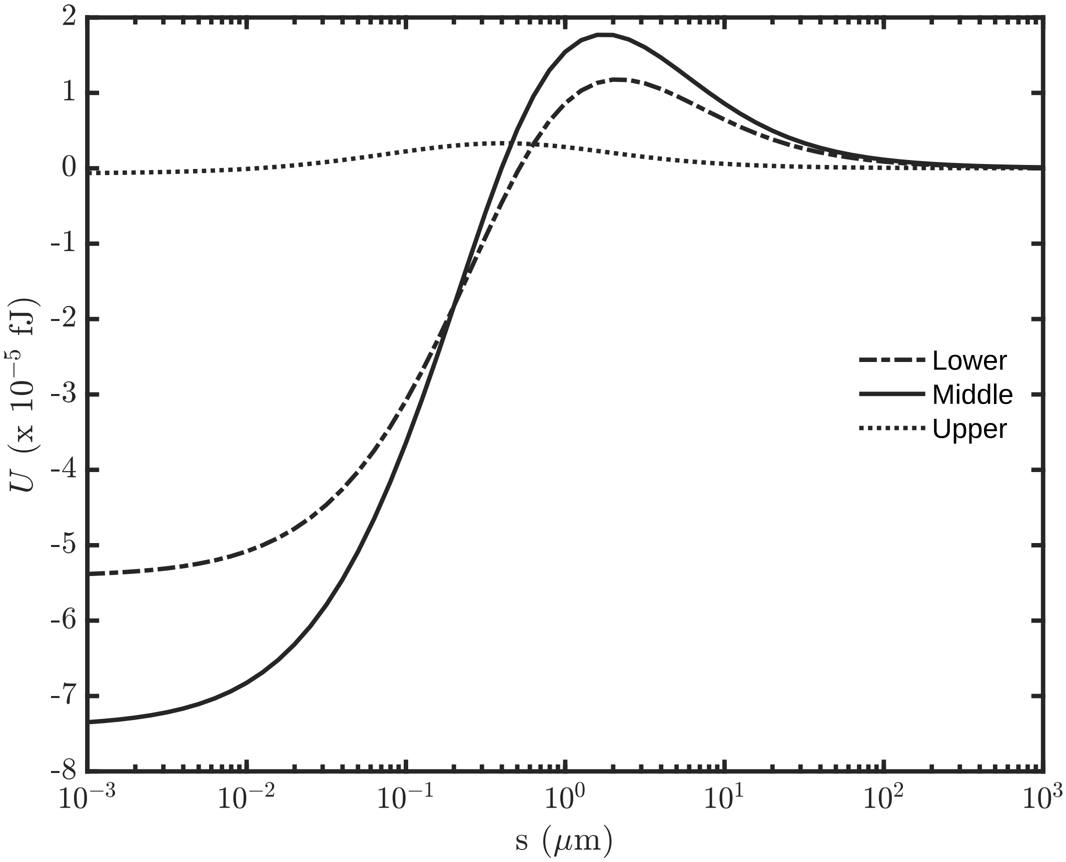

Figure S2: Profiles of the electrostatic interaction energy, U, against the surface-to-surface separation, s, for two like-charged Venusian cloud particles in each cloud layer.

Table S1: A table summarizing the key electrostatic parameters for two interacting like-charged particles calculated for a range of sizes, r, and charges, q, across the main Venusian cloud deck, including the Coulomb barrier height, E_{Coul}, the contact point energy, E_0 and the percentage of particles that have sufficient velocity to aggregate, i.e. the aggregation percentage.

Cloud Region	Altitude (km)	Temp. (K)	Charge (e)	Radius (μm)		E_{Coul} (x 10^{-5} fJ)	E_0 (x 10^{-5} fJ)	Agg. %
				r_1	r_2			
Upper	70	238	-5.5	0.2	1.0	0.279	-0.175	31.11
	65.5	237	-13.5	0.2	1.0	1.682	-1.053	2.074
	61	248	-21.5	0.2	1.0	4.266	-2.672	0.002
Middle	56.5	284	-31.5	0.15	3.5	2.617	-29.92	0.410
		284	-31.5	0.15	1.25	7.324	-19.44	0.000
		284	-31.5	1.25	3.5	2.613	1.487	0.408
	54.5	302	-34	0.15	3.5	3.049	-34.85	0.226
		302	-34	0.15	1.25	8.533	-22.65	0.000
		302	-34	1.25	3.5	3.044	1.732	0.230
	52.5	322	-28	0.15	3.5	2.068	-23.64	2.625
		322	-28	0.15	1.25	5.787	-15.36	0.001
		322	-28	1.25	3.5	2.065	1.175	1.855
Lower	50.5	340	-22.5	0.2	4.0	1.163	-11.12	18.06
		340	-22.5	0.2	1.0	4.672	-2.926	0.024
		340	-22.5	1.0	4.0	1.166	-0.067	15.72
	49	354	-23.5	0.2	4.0	1.268	-12.21	16.35
		354	-23.5	0.2	1.0	5.097	-3.192	0.016
		354	-23.5	1.0	4.0	1.272	0.072	14.43
	47.5	367	-25	0.2	4.0	1.435	-13.82	13.36
		367	-25	0.2	1.0	5.768	-3.612	0.007
		367	-25	1.0	4.0	1.440	0.082	12.58

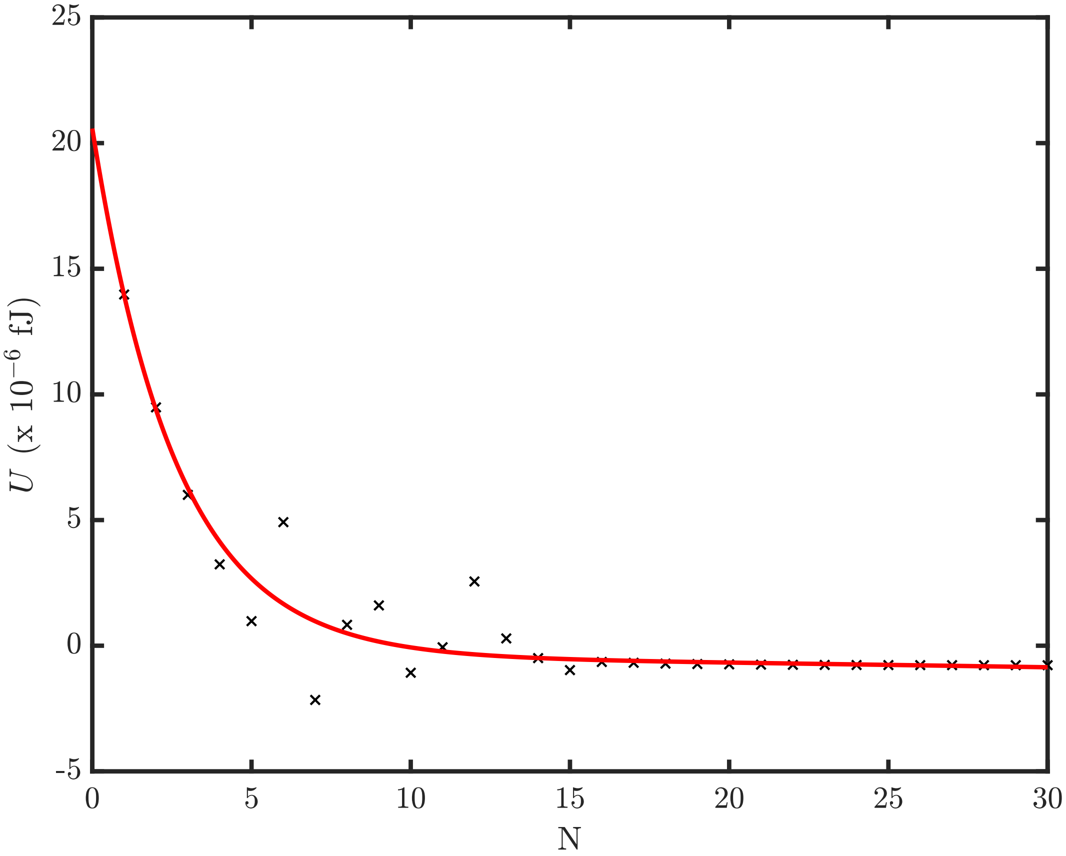

Figure S3: Convergence of the electrostatic interaction energy, U, as computational expense increases, i.e. as the number of terms, N, used in the multipolar expansion increases, for two like-charged, polarizable particles (r_1 = 4.0 μm, q_1 = -23 e; r_2 = 0.2 μm, q_2 = -22 e; k = 100) as found in Venus' lower cloud layer, with surface-to-surface separation s = 0.5 µm. The convergence is shown by the fitted curve (solid red line) alongside the calculated results (black markers).
